# A machine learning approach towards assessing consistency and reproducibility: an application to graft survival across three kidney transplantation eras

**DOI:** 10.3389/fdgth.2024.1427845

**Published:** 2024-09-03

**Authors:** Okechinyere Achilonu, George Obaido, Blessing Ogbuokiri, Kehinde Aruleba, Eustasius Musenge, June Fabian

**Affiliations:** ^1^ Division of Epidemiology and Biostatistics, School of Public Health, University of the Witwatersrand, Johannesburg, South Africa; ^2^ Center for Human-Compatible Artificial Intelligence (CHAI), Berkeley Institute for Data Science (BIDS), University of California, Berkeley, Berkeley, CA, United States; ^3^ Department of Computer Science, Brock University, St. Catharines, ON, Canada; ^4^ School of Computing and Mathematical Sciences, University of Leicester, Leicester, United Kingdom; ^5^ Wits Donald Gordon Medical Centre, Faculty of Health Sciences, School of Clinical Medicine, University of the Witwatersrand, Johannesburg, South Africa

**Keywords:** kidney transplant, immunosuppressive regimen, transplantation era, graft survival, machine learning, reproducibility, consistency

## Abstract

**Background:**

In South Africa, between 1966 and 2014, there were three kidney transplant eras defined by evolving access to certain immunosuppressive therapies defined as *Pre-CYA* (before availability of cyclosporine), *CYA* (when cyclosporine became available), and *New-Gen* (availability of tacrolimus and mycophenolic acid). As such, factors influencing kidney graft failure may vary across these eras. Therefore, evaluating the consistency and reproducibility of models developed to study these variations using machine learning (ML) algorithms could enhance our understanding of post-transplant graft survival dynamics across these three eras.

**Methods:**

This study explored the effectiveness of nine ML algorithms in predicting 10-year graft survival across the three eras. We developed and internally validated these algorithms using data spanning the specified eras. The predictive performance of these algorithms was assessed using the area under the curve (AUC) of the receiver operating characteristics curve (ROC), supported by other evaluation metrics. We employed local interpretable model-agnostic explanations to provide detailed interpretations of individual model predictions and used permutation importance to assess global feature importance across each era.

**Results:**

Overall, the proportion of graft failure decreased from 41.5% in the *Pre-CYA* era to 15.1% in the *New-Gen* era. Our best-performing model across the three eras demonstrated high predictive accuracy. Notably, the ensemble models, particularly the Extra Trees model, emerged as standout performers, consistently achieving high AUC scores of 0.95, 0.95, and 0.97 across the eras. This indicates that the models achieved high consistency and reproducibility in predicting graft survival outcomes. Among the features evaluated, recipient age and donor age were the only features consistently influencing graft failure throughout these eras, while features such as glomerular filtration rate and recipient ethnicity showed high importance in specific eras, resulting in relatively poor historical transportability of the best model.

**Conclusions:**

Our study emphasises the significance of analysing post-kidney transplant outcomes and identifying era-specific factors mitigating graft failure. The proposed framework can serve as a foundation for future research and assist physicians in identifying patients at risk of graft failure.

## 1 Introduction

Kidney transplantation is the standard of care for the management of kidney failure, significantly enhancing quality of life and increasing longevity compared to the alternative, which is chronic dialysis treatment (1–5). Nonetheless, a kidney transplant’s long-term success relies on the transplanted organ’s survival, known as graft survival. Over the years, considerable progress has been made regarding maintenance immunosuppression regimens to improve graft survival post-kidney transplant (6–8). In Johannesburg, three eras of post-kidney transplant maintenance immunosuppression therapy are described (i) from 1966 to 1983 using combined azathioprine and cortisone; (ii) from 1983 to 2000 replacing azathioprine with cyclosporine; and (iii) starting in 2001, the introduction of sirolimus, everolimus, and mycophenolate mofetil (9–11). These advancements in immunosuppressive therapy have improved graft survival rates over the years. (12, 13). Identifying prognostic factors contributing to graft failure could inform the post-kidney transplant management of recipients to improve long-term graft survival.

Globally, and in South Africa, preserving the long-term survival of the graft after kidney transplant is the ultimate goal, not only for the enhanced survival benefits and improved quality of life of the recipient but also because organ donor shortages persist. In the event of graft failure, maintenance dialysis must be re-initiated, and re-transplantation must be considered with adverse consequences for the patient and a disproportionate increase in the cost of care (when compared with the cost of maintenance immunosuppression therapy) (14–16). Studies have identified donor-related and recipient-related factors that impact kidney transplant outcomes. More specifically, previous research in South Africa has shown the impact of donor type, delayed graft function, recipient age, and self-reported ethnicity on graft survival based on univariate and multivariate survival models (10, 13, 17, 18). These earlier studies have significantly contributed to transplantation outcomes in African settings. However, they are primarily based on conventional statistical methods. While traditional statistical methods can offer insights into how prognostic factors influence survival, some approaches used in these previous studies may not provide a realistic representation of real-life situations when identifying factors influencing outcome (19). Furthermore, many of these studies are constrained by complete case analysis or the exclusion of important variables due to missing information, and none of these studies considered exploring graft survival across the three eras (10, 13, 18).

Medical research studies have extensively employed machine learning (ML) to enhance predictive risk assessment, resulting in more accurate predictions (20–25). This approach can assist physicians in risk assessment by identifying patients who might be at a higher risk of graft failure following kidney transplantation. In recent years, ML models have gained increasing attention in medical research for developing diagnostic and predictive models for medical outcomes (21, 26–29). These ML models have also been successfully used in kidney transplant studies and have demonstrated good performance in predicting graft survival at different survival times (30–33). For example, Moghadam and Ahmadi (34) developed a clustering method using the Red Deer Algorithm (RDA), together with other ML classification algorithms and proposed a three-stage clustering-based undersampling approach to better handle class imbalances. Topuz et al. (35) designed a method that combines the Bayesian belief network algorithm, feature selection, and multiple ML techniques to predict kidney graft survival using data from over 31,000 U.S. patients. The study suggests that this approach can be applied to other transplant datasets. Fabreti-Oliveira et al. (36) employed two gradient boosting algorithms to analyse data from 627 kidney transplant patients and identified that serum creatinine levels at discharge, pre-transplant weight and age were key factors affecting early graft loss. The study highlights the potential of ML for informed decision-making in transplantation. Although ML has not been utilised to pinpoint significant prognostic factors in South African transplant units, there are other knowledge gaps concerning previously developed models. Most of these models have yet to be validated outside of the study cohort in which they were developed. It has been observed that many ML models are constrained by factors such as geographical location, study interval, historical period, and methodological approach (20, 28, 37). Hence, these models may not generalise well when transported to patients with dissimilar characteristics compared to those used to develop the model.

In this study, we developed and validated ML models to predict 10-year graft survival using clinical and socio-demographic characteristics of kidney transplant recipients and their donors in Johannesburg between 1966 and 2014 – covering three eras of maintenance immunosuppression regimens. Our era-based models were designed to examine the risk factors associated with each transplant era and to gauge the ML algorithms’ discriminative capability between outcome classes, namely graft failure or survival. By focusing on era-specific models, we ensured that our findings are consistent (stable and reliable) and reproducible (valid and replicable) across different historical contexts, capturing variations in risk factors and outcomes. Additionally, we tested the transportability of our models by developing them using data from one era and validating them in another era, highlighting the difficulty of applying ML models across different settings and emphasizing the need for tailored approaches. This research is relevant because it is region-specific, addressing challenges such as limited access to organ transplant facilities and improving kidney transplant graft survival outcomes in resource-limited settings countries. Finally, the developed models will serve as the foundation for future model development and external validation within and beyond the study area.

The rest of this paper is organised as follows. In the subsequent section, we present the design for this study, followed by descriptions of the algorithms. Section 3 showcases the study’s results, and Section 6 presents a comprehensive discussion of the results and highlights avenues for future research.

## 2 Materials and methods

In this section, we offer insights into the dataset, starting with our approach to data acquisition. We then transition into the methods used for data preprocessing and developing ML models. Next, we present LIME, an explainability ML method used to interpret and understand the predictions made by the predictive models. Finally, we apply permutation feature importance to evaluate and rank the contribution of each feature to the model’s predictions.

### 2.1 Study design and population

**Figure 1:**
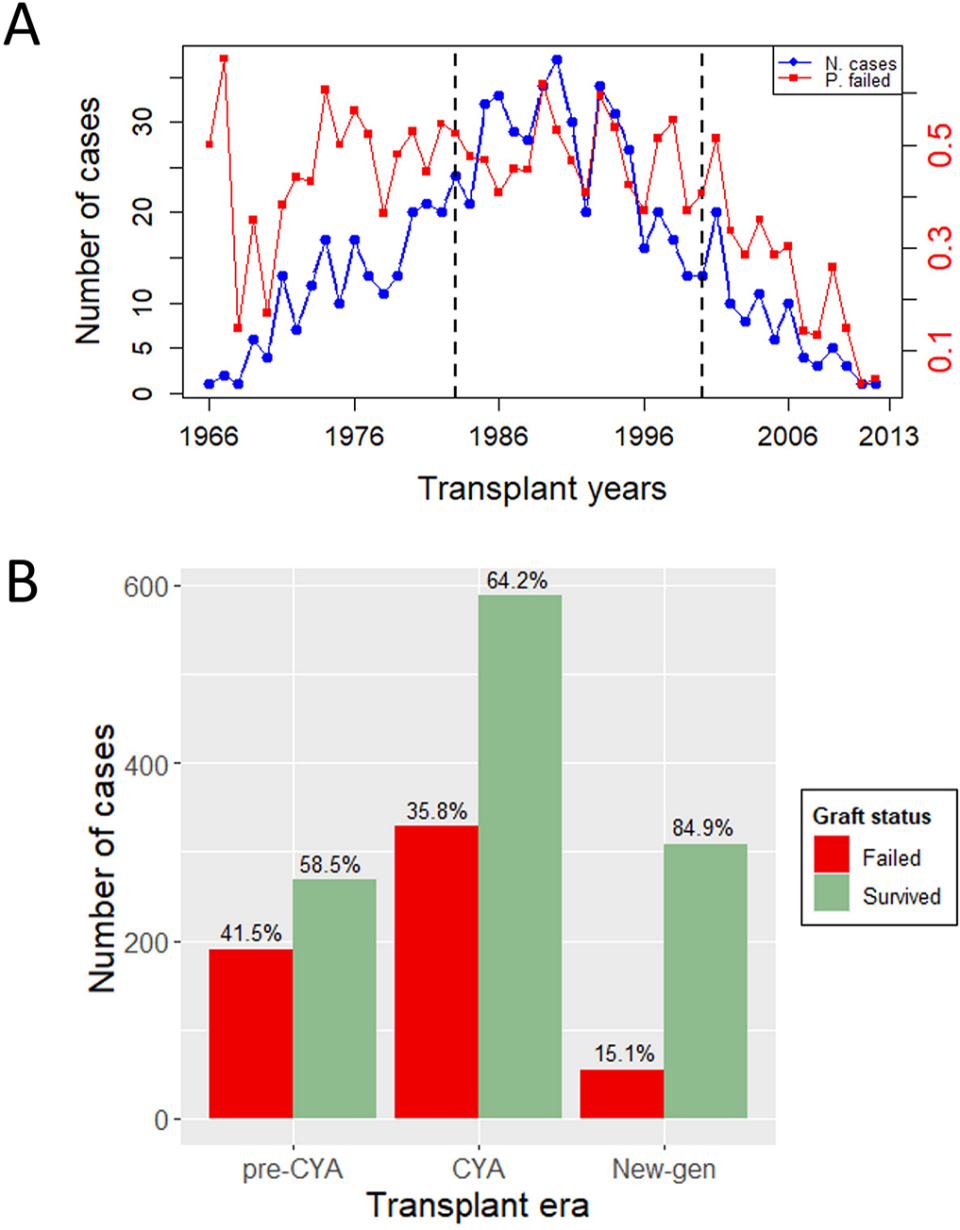
Outcome variable description. **(A)** A Time series plot showing the number of transplant cases and proportion of graft failure in the three eras and over the study period. The dashed lines show the study period for *pre-CYA*, *CYA* and *New-GEN*. **(B)** A barplot illustrates the number of transplant cases and the distribution of graft failure across the transplant era.

This study is a retrospective analysis of patients who underwent kidney transplants at Charlotte Maxeke Johannesburg Academic Hospital (CMJAH), South Africa, from 1966 to 2014. This time frame encompasses three distinct immunosuppressive eras: the pre-cyclosporine era (1966–1983), the cyclosporine era (1984–2000), and the new-generation era (2001–2014). For easy mentioning of the eras, as we advance in this study, we have denoted these eras as *pre-CYA*, *CYA* and *new-GEN*. Ethical approval for this study was received from the University of Witwatersrand Faculty of Health Sciences Research Ethics Committee [M121186]. More details of this study can be seen in previous studies, including Pitcher et al. (11), Fabian et al. (18). The analysis was limited to patients aged 18 or above who received their first kidney transplant and were followed up for at least one year after surgery. Patients were excluded if the exact age at the transplant or date of transplant was unknown. The final dataset contains 1,738 cases, including 458, 916 and 364 transplant cases in the *pre-CYA*, *CYA* and *New-GEN* eras. Figure 1A shows the number of transplant cases and the proportion of graft failure across the three immunosuppressive eras. The trend in this plot suggests that the number of kidney transplant cases was highest during the *CYA* era compared with other eras. The highest and lowest proportions of graft failure were observed in *pre-CYA* and *New-GEN* eras, respectively. The number of recipients with a failed graft ten years post-kidney transplant is lower than those whose graft survived, with a proportion of 41.5%, 35.8% and 15.1%, respectively, across the three eras (Figure 1B).

### 2.2 Transplant overview

The dataset encompasses a total of 1,738 kidney transplant records, split into three distinct eras: *pre-CYA* (458 entries), *CYA* (916 entries), and *New-GEN* (364 entries), as summarised in Table 1. The median recipient age was approximately 38 (18–68) years, with a slight variation among the eras, as *New-gen* recipients tended to be a bit older than recipients in other eras. Overall, the median age of the recipients who experienced graft failure is higher than that of those who did not experience graft failure. The overall median donor age was 27 (1–72) years. For the recipient with a failed graft, the median age of the kidney donor was 28, while the median donor age for those who did not experience graft failure was 25 years. The Mann–Whitney U test showed that recipient and donor age significantly differed between recipients who experienced graft failure and those who did not experience graft failure. Approximately 84% of these patients received a kidney from deceased donors. Regarding self-reported ethnicity, most recipients were White, followed by Black and other ethnicities. Primary glomerular disease emerged as the predominant cause of end-stage kidney disease, especially in the *pre-CYA* era. The *New-GEN* era was marked by a higher prevalence of hypertension as a cause. Treatment-wise, methylprednisolone as an induction therapy was predominant in the *New-GEN* era. A notable number of patients experienced delayed graft function, with the *CYA* era having the most observed cases. The Chi-square test showed significant associations between graft survival status and variables, including donor type and delayed graft function, acute rejection and chronic rejection at a 5% significance level.

**Table 1:** Demographic and clinical characteristics of study participants across the transplant eras.

Patient characteristics (n[%] unless otherwise indicated)		Pre-CYA Graft status		CYA Graft status		New-Gen Graft status		Overall Graft status		p-value
		Failed (N=268)	Survived (N=190)	Failed (N=588)	Survived (N=328)	Failed (N=309)	Survived (N=55)	Failed	Survived	
Donor type										
	Deceased	213 (79.5)	175 (92.1)	484 (82.3)	296 (90.2)	232 (75.1)	50 (90.9)	929 (79.7)	521 (90.9)	<0.001
	Living	54 (20.1)	15 (7.9)	103 (17.5)	31 (9.5)	76 (24.6)	4 (7.3)	233 (20.0)	50 (8.7)	
	Missing	1 (0.4)	0 (0)	1 (0.2)	1 (0.3)	1 (0.3)	1 (1.8)	3 (0.3)	2 (0.3)	
Donor age (median[range])		24.0 [5.00, 63.0]	25.0 [4.00, 63.0]	27.0 [1.00, 65.0]	24.0 [1.0, 61.0]	32.0 [1.0, 72.0]	43.0 [5.0, 72.0]	28.0 [1.0, 72.0]	25.0 [1.0, 72.0]	<0.001
	Missing	33 (12.3)	25 (13.2)	50 (8.5)	29 (8.8)	32 (10.4)	6 (10.9)	115 (9.9)	60 (10.5)	
Recipient age (median[range])		35.0 [18.0, 65.0]	40.0 [19.0, 58.0]	36.0 [18.0, 63.0]	41.0 [18.0, 68.0]	39.0 [18.0, 66.0]	47.0 [19.0, 64.0]	37.0 [18.0, 66.0]	41.0 [18.0, 68.0]	<0.001
Donor-recipient sex match										
	No	104 (38.8)	77 (40.5)	261 (44.4)	144 (43.9)	138 (44.7)	22 (40.0)	503 (43.2)	243 (42.4)	0.955
	Yes	144 (53.7)	89 (46.8)	297 (50.5)	170 (51.8)	140 (45.3)	25 (45.5)	581 (49.9)	284 (49.6)	
	Missing	20 (7.5)	24 (12.6)	30 (5.1)	14 (4.3)	31 (10.0)	8 (14.5)	81 (7.0)	46 (8.0)	
Recipient ethnicity										
	Black	6 (2.2)	7 (3.7)	148 (25.2)	123 (37.5)	203 (65.7)	46 (83.6)	357 (30.6)	176 (30.7)	0.791
	Others	32 (11.9)	15 (7.9)	78 (13.3)	49 (14.9)	42 (13.6)	4 (7.3)	152 (13.0)	68 (11.9)	
	White	227 (84.7)	168 (88.4)	362 (61.6)	153 (46.6)	64 (20.7)	5 (9.1)	653 (56.1)	326 (56.9)	
	Missing	3 (1.1)	0 (0)	0 (0)	3 (0.9)	0 (0)	0 (0)	3 (0.3)	3 (0.5)	
Donor-recipient blood match										
	No	53 (19.8)	43 (22.6)	63 (10.7)	27 (8.2)	27 (8.7)	4 (7.3)	143 (12.3)	74 (12.9)	0.862
	Yes	195 (72.8)	139 (73.2)	514 (87.4)	292 (89.0)	255 (82.5)	49 (89.1)	964 (82.7)	480 (83.8)	
	Missing	20 (7.5)	8 (4.2)	11 (1.9)	9 (2.7)	27 (8.7)	2 (3.6)	58 (5.0)	19 (3.3)	
Delayed graft function										
	No	152 (56.7)	89 (46.8)	361 (61.4)	204 (62.2)	230 (74.4)	36 (65.5)	743 (63.8)	329 (57.4)	0.007
	Yes	107 (39.9)	90 (47.4)	214 (36.4)	118 (36.0)	62 (20.1)	19 (34.5)	383 (32.9)	227 (39.6)	
	Missing	9 (3.4)	11 (5.8)	13 (2.2)	6 (1.8)	17 (5.5)	0 (0)	39 (3.3)	17 (3.0)	
Diabetes mellitus										
	No	236 (88.1)	167 (87.9)	543 (92.3)	296 (90.2)	277 (89.6)	47 (85.5)	1,056 (90.6)	510 (89.0)	0.097
	Yes	20 (7.5)	9 (4.7)	32 (5.4)	28 (8.5)	15 (4.9)	8 (14.5)	67 (5.8)	45 (7.9)	
	12 (4.5)	14 (7.4)	13 (2.2)	4 (1.2)	17 (5.5)	0 (0)	42 (3.6)	18 (3.1)		
cause of KF (primary renal)										
	No	81 (30.2)	50 (26.3)	318 (54.1)	201 (61.3)	247 (79.9)	46 (83.6)	646 (55.5)	297 (51.8)	0.170
	Yes	187 (69.8)	140 (73.7)	270 (45.9)	127 (38.7)	62 (20.1)	9 (16.4)	519 (44.5)	276 (48.2)	
cause of KF (hypertension-associated)										
	No	254 (94.8)	185 (97.4)	422 (71.8)	207 (63.1)	137 (44.3)	18 (32.7)	813 (69.8)	410 (71.6)	0.482
	Yes	14 (5.2)	5 (2.6)	166 (28.2)	121 (36.9)	172 (55.7)	37 (67.3)	352 (30.2)	163 (28.4)	
cause of KF (urological)										
	No	234 (87.3)	180 (94.7)	537 (91.3)	310 (94.5)	295 (95.5)	53 (96.4)	1,066 (91.5)	543 (94.8)	0.019
	Yes	34 (12.7)	10 (5.3)	51 (8.7)	18 (5.5)	14 (4.5)	2 (3.6)	99 (8.5)	30 (5.2)	
cause of KF (inherited)										
	No	253 (94.4)	174 (91.6)	522 (88.8)	307 (93.6)	295 (95.5)	55 (100)	1,070 (91.8)	536 (93.5)	0.246
	Yes	15 (5.6)	16 (8.4)	66 (11.2)	21 (6.4)	14 (4.5)	0 (0)	95 (8.2)	37 (6.5)	
Surgical complication	Yes	126 (47.0)	114 (60.0)	222 (37.8)	125 (38.1)	141 (45.6)	22 (40.0)	489 (42.0)	261 (45.5)	0.172
	No	142 (53.0)	76 (40.0)	366 (62.2)	203 (61.9)	168 (54.4)	33 (60.0)	676 (58.0)	312 (54.5)	
Egfr (median[range])	63.8 [9.73, 249]	55.5 [11.7, 208]	56.8 [3.40, 123]	54.6 [6.87, 179]	60.9 [6.4, 168]	44.8 [26.4, 113]	59.4 [3.4, 249]	54.0 [6.9, 208]		
	Missing	85 (31.7)	78 (41.1)	171 (29.1)	130 (39.6)	123 (39.8)		379 (32.5)	230 (40.1)	0.005
Induction therapy (Methylprednisolone)										
	No	37 (13.8)	32 (16.8)	71 (12.1)	34 (10.4)	27 (8.7)	4 (7.3)	135 (11.6)	70 (12.2)	0.762
	Yes	231 (86.2)	158 (83.2)	517 (87.9)	294 (89.6)	282 (91.3)	51 (92.7)	1,030 (88.4)	503 (87.8)	
Acute rejection diagnosis (biopsy)										
	No	238 (88.8)	171 (90.0)	498 (84.7)	297 (90.5)	260 (84.1)	41 (74.5)	996 (85.5)	509 (88.8)	0.065
	Yes	30 (11.2)	19 (10.0)	90 (15.3)	31 (9.5)	49 (15.9)	14 (25.5)	169 (14.5)	64 (11.2)	
Chronic rejection diagnosis (biopsy)										
	No	250 (93.3)	182 (95.8)	522 (88.8)	311 (94.8)	251 (81.2)	49 (89.1)	1,023 (87.8)	542 (94.6)	<0.001
	Yes	18 (6.7)	8 (4.2)	66 (11.2)	17 (5.2)	58 (18.8)	6 (10.9)	142 (12.2)	31 (5.4)	
Rejection treatment (IVI steroid pulse)										
	No	76 (28.4)	50 (26.3)	236 (40.1)	118 (36.0)	280 (90.6)	45 (81.8)	592 (50.8)	213 (37.2)	
	Yes	192 (71.6)	140 (73.7)	352 (59.9)	210 (64.0)	29 (9.4)	10 (18.2)	573 (49.2)	360 (62.8)	<0.001

### 2.3 Data pre-processing

In this study, graft survival is the time from transplant to failure of the graft, defined as the earliest time to return to dialysis. Death with a functioning graft and a few patients lost to follow-up were censored based on the time of death or date last seen. A patient graft was classified as “graft failure” if the graft had been recorded as failed in the database, as per the definition above; otherwise, the graft status was classified as “survived”. Information relating to patients who underwent more than one transplant was excluded from this study; in other words, the scope of this study was restricted to first graft failure.

We retrieved 1,207 pre-, peri and post-transplantation information from the database. The pre-transplant measures include the cause of kidney failure (KF), donor type, donor and recipient sex and blood group, recipient age, recipient self-reported ethnicity, and donor age, as shown in Table 1. The peri-transplant features are those measured during the transplant, which are estimated glomerular filtration rate and Induction therapy. The post-transplantation characteristics considered in this study are delayed graft function (DGF), surgical complications, biopsy-proven acute or chronic rejection, and rejection treatment. Repeated information measured months or years post-transplant was dropped from the analysis. We also dropped features with empty records and variables relating to data-capturing details alone. Only 39 features measured across the three transplant eras were extracted for pre-processing.

Descriptive statistics and data visualisation were used to assess the data quality and understand the study features’ patterns and relationships. Overall, approximately 1% of the case records are missing in the dataset, which was contributed by eight features, including systolic blood pressure at transplant, diabetes at transplant and delayed graft function. To render the data more applicable to this study, we have addressed the problem of missingness using the missForest imputation algorithm, which uses a random forest approach to predict missing values in a database. missForest is an ML technique that has shown good performance in predicting missing values in mixed data types across different fields of study (38, 39) and performs better than other imputation methods.

Feature engineering was conducted by grouping each feature category with low frequency with their related category. For instance, donor type original class “deceased”, “living related”, and “living unrelated” donors were recategorised as “deceased” and “living” donors. This addresses the problem of representativeness of each factor variable category and enables the model to sufficiently learn from each feature category to improve each feature discriminative power and avoid bias in prediction. Donor and recipient blood groups were matched to create a single variable “donor-recipient blood group match”. Also, the donor and recipient sex were matched to generate the “donor-recipient sex match” variable.

### 2.4 The kidney transplant analysis

**Figure 2:**
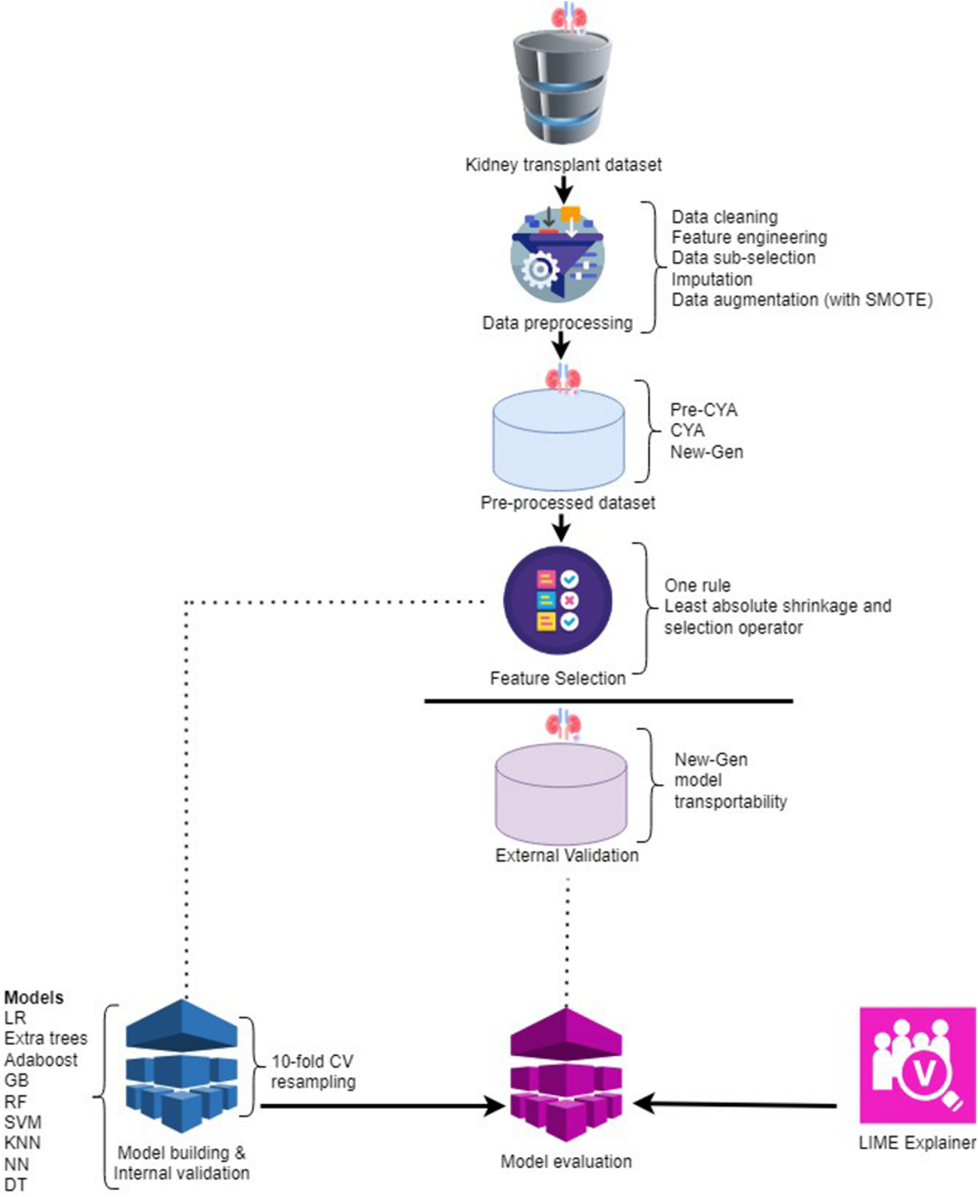
The framework of the kidney transplant analysis.

The process of kidney transplant analysis is a multifaceted procedure with several steps, each playing a pivotal role in generating meaningful insights. It begins with data preprocessing as presented in Figure 2. During this stage, the raw data undergoes various transformations. Techniques such as data cleaning, feature engineering, data sub-selection, imputation, and applying the Synthetic Minority Over-sampling Technique (SMOTE) are utilised. These methods collectively work towards refining the data, eliminating noise and irrelevant information, addressing missing values, and achieving a balanced dataset.

After preprocessing, the data is organised into three distinct categories: *pre-CYA*, *CYA*, and *New-Gen*. This division is crucial for the following phases of feature selection and model construction. Three feature selection techniques were employed: the One-Rule, Random Forest and the Least Absolute Shrinkage and Selection Operator (LASSO). These techniques aid in pinpointing the most significant features from the data, which have the utmost predictive power for the target variable.

We utilised internal and external validation techniques during the study to ensure our models’ robustness and generalisability. Internal validation, often termed “resampling” validation, refers to the process of evaluating the model’s performance on a subset of the training data. This is typically achieved using techniques like k-fold cross-validation, where the data is partitioned into “k=10” subsets. The model is trained on k−1 of these subsets and tested on the remaining one. This process is repeated k times, each subset serving as the test set once. The primary advantage of internal validation is that it provides a more robust estimate of the model’s performance, minimising the risk of overfitting by ensuring the model performs well across multiple, varied subsets of the training data. This stage encompasses utilising various ML models, such as Logistic Regression, Extra Trees, Adaboost, Gradient Boosting, Random Forest, Support Vector Machine, K-nearest neighbours, Neural Network, and Decision Tree.

After the models’ construction and internal validation are assessed on an external validation dataset. External validation, on the other hand, assesses the model’s performance on an entirely separate dataset that it has never seen during training. This dataset is not used in any phase of the model-building process. The essence of external validation is to gauge the model’s real-world applicability and its potential performance on new, unseen data.

Once the models are built and internally validated, they seem like black boxes, making predictions that are hard to understand. Imagine a complex model that predicts whether a kidney transplant will be successful or not. It considers numerous factors, such as the donor’s age, the compatibility of donor and recipient, the health of the recipient, and many others. However, once the prediction is made, it is not immediately clear which factors were most influential in making that prediction. This is problematic because clinicians and patients might need to understand the rationale behind the prediction to make informed decisions. LIME addresses this issue by approximating the complex model with a simpler, interpretable model (e.g., a logistic regression model) locally around the prediction (40). This simpler model can then be studied to understand how each feature influences the prediction. For example, the LIME explanation might reveal that the model predicted a high chance of transplant success mainly because the donor and recipient were highly compatible and the recipient was in good health.

In the subsequent section, a comprehensive discussion on the various models utilised for this research is provided. This includes an explanation of the functioning of each model. A thorough understanding of the models is indispensable for accurately interpreting the results and making well-informed decisions based on the analysis.

### 2.5 Machine learning classification models

In this section, we describe the ML models specifically tailored for the classification tasks used for this study.

#### 2.5.1 AdaBoost

In our study, we applied a technique called AdaBoost to enhance the performance of our machine learning model. Adaptive Boosting, short for AdaBoost, is an ensemble learning algorithm designed to enhance the performance of ML models (41, 42). According to Freund and Schapire (43), AdaBoost works by combining several simple models, known as weak learners, into a single, more accurate model. Each weak learner is trained on our dataset and contributes to the final prediction. Initially, all data points in our dataset were treated equally. As we trained each weak learner, we paid more attention to the examples that were difficult to classify correctly. This means that the model focused on getting the hard cases right.

We repeated this process for multiple iterations, adjusting the importance of each data point based on the previous models’ performance. Misclassified examples were given more weight, so the next weak learner would focus more on them. After several rounds, we combined the weak learners into a single strong model. Each weak learner had a say in the final prediction, but the more accurate learners had a bigger influence. By using AdaBoost, we were able to create a model that performed better on our dataset compared to using just a single simple model. This approach helped us achieve more accurate and reliable results.

#### 2.5.2 Extreme gradient boosting

We applied extreme gradient boosting or XGBoost to our dataset to enhance the accuracy and efficiency of our machine learning model. XGBoost is an advanced ensemble technique that combines the predictions of multiple models to produce a more accurate final prediction (25, 44). We started by dividing our dataset into training and testing sets, using the training set to build the model and the testing set to evaluate its performance. XGBoost iteratively trained a series of decision trees on the training set, with each tree focusing on correcting the errors made by the previous ones. This iterative process continuously improved the model’s accuracy.

XGBoost’s versatility allowed us to handle different types of prediction tasks, such as regression and classification, by using appropriate loss functions for each task. For instance, we used squared error loss for regression and logistic loss for classification. Additionally, XGBoost includes a regularization term that penalizes overly complex models to prevent overfitting. This term considers the number of terminal nodes in the trees and the scores assigned to these nodes. By applying XGBoost, we created a robust model that accurately captured the patterns in our data, significantly improving the model’s performance and reliability for our prediction tasks.

#### 2.5.3 Random forest

We utilized the Random Forest algorithm to analyze our dataset. Random Forest is an ensemble learning method that constructs multiple decision trees to perform both classification and regression tasks (29, 45, 46). For classification, it predicts the class that is chosen by the majority of the trees, and for regression, it averages the predictions of all the trees. This technique helps to address the problem of overfitting often encountered with individual decision trees. Random Forest works by creating each tree from a different bootstrap sample of the data, and generally, increasing the number of trees enhances the accuracy of the model (29). Additionally, Random Forest performs automatic feature selection, which improves the performance of traditional decision tree algorithms (47).

In applying Random Forest to our dataset, we divided the data into training and testing sets. The algorithm built numerous decision trees using different subsets of the training data. For classification tasks, the final prediction was determined by the majority vote from all the trees, while for regression tasks, the average prediction of all the trees was used. This approach not only improved the accuracy of our model but also made it more robust and less prone to overfitting. The ability of Random Forest to automatically select relevant features further enhanced the efficiency and effectiveness of our analysis, leading to more reliable and interpretable results.

#### 2.5.4 Decision trees

Decision Trees are a widely used supervised learning method for classification and regression tasks (48, 49). They work by creating a model that predicts the value of a target variable using simple decision rules derived from the data’s features. The core idea is to split the dataset into subsets based on specific criteria, ensuring that each split results in more homogeneous subsets (50). This splitting process continues until the model can make accurate predictions. Decision Trees rely on various metrics to determine the best splits, such as entropy, information gain, and Gini impurity. These metrics measure the disorder or impurity within the data and help guide the tree-building process to create effective and accurate models.

We used Decision Trees to analyze our dataset by dividing it into training and testing sets. The Decision Tree algorithm built the model by learning decision rules from the training data, using metrics like entropy to determine the best splits. For instance, entropy measures the randomness or unpredictability in the data, and information gain represents the reduction in entropy after a split. Gini impurity, another metric, quantifies how often a randomly selected item would be incorrectly classified. By applying these metrics, the Decision Tree algorithm iteratively split the data into smaller, more uniform subsets, leading to a model that could accurately predict outcomes. This method provided a clear and interpretable structure for understanding the relationships in our data, making it a valuable tool for our analysis.

#### 2.5.5 Extra trees

Extra Trees, also regarded as *Extremely Randomised Trees*, is an ensemble method designed for supervised classification and regression tasks (51). As an ensemble method, the Extra Trees introduce a higher level of randomness in the tree-building process (52). Unlike traditional tree methods that identify the optimal decision split for a given attribute, Extra Trees randomises the choice of attributes and their respective cut points. This double layer of randomness—both in attribute and split point selection—often results in a more diversified set of base trees, which can enhance the model’s generalisation capabilities.

This method can sometimes outperform more deterministic algorithms, especially in scenarios with a lot of noise. In the Extra Trees Classifier, decision trees are utilised. The parameter k determines the number of features selected in a random sample from the feature set.

To apply Extra Trees to our dataset, we divided the data into training and testing sets. The algorithm then constructed numerous decision trees using random subsets of features and split points for each tree. This process involves training the model with these randomly generated trees and combining their predictions to produce a final outcome. By averaging the predictions of all the trees in the ensemble, Extra Trees provided a more stable and accurate model. This approach allowed us to capture complex patterns in the data and make reliable predictions, enhancing the overall performance of our analysis.

#### 2.5.6 Logistic regression

Logistic regression was applied to our dataset to evaluate the relationship between a categorical outcome variable and multiple predictor variables (21, 53, 54). This method is particularly useful for binary classification tasks, where the goal is to predict one of two possible outcomes (27, 55, 56). In our analysis, logistic regression was used to model the likelihood of a specific outcome based on various input features.

To implement logistic regression on our dataset, we first identified the target variable and the predictor variables. The model was then trained using the data to find the best-fit logistic curve, which represents the probability of the target variable occurring given the predictor variables. This approach allowed us to make predictions about the categorical outcome, providing insights into how different factors influence the likelihood of the outcome in our dataset.

#### 2.5.7 Support vector machine

Support Vector Machine (SVM) is a supervised learning technique used for both classification and regression tasks. Its primary goal is to find the best boundary that separates different classes in the dataset. This boundary, known as the decision boundary, is determined by maximizing the margin between the closest data points from each class, ensuring that the model can effectively distinguish between positive and negative instances (57, 58). SVM is particularly effective when the data is linearly separable, meaning there is a clear dividing line between the classes.

In our dataset, SVM was applied to classify instances based on the features provided. By training the SVM model on the dataset, we identified the optimal decision boundary that separates different classes. This boundary was used to predict the class labels for new data points, helping us understand how the features influence the classification outcome. The SVM model’s ability to maximize the margin between classes contributed to its effectiveness in accurately classifying the data in our analysis.

#### 2.5.8 K-nearest neighbour

K-Nearest Neighbour (KNN) is a simple and widely used classification algorithm that works on the idea that similar data points are close to each other in the feature space. This makes it effective for tasks where it’s difficult to describe the relationship between features and outcomes using more complex models. KNN is commonly applied in areas like image recognition, recommendation systems, and medical diagnosis (59–61). In our dataset, we used KNN to classify data points by identifying the ’k’ closest neighbors to each point and predicting its class based on the majority class among these neighbors. This approach allowed us to classify new data based on the patterns observed in the nearest existing data points.

#### 2.5.9 MLP neural network

The Multilayer Feedforward Perceptron (MLP) is a popular neural network architecture used for tasks like classification and regression. It consists of layers of neurons where each neuron is connected to all neurons in the next layer, with no connections within the same layer. During training, the network adjusts its weights and biases to minimize the difference between the predicted and actual outcomes (62, 63). In our dataset, we applied MLP to model complex relationships between features and the target variable by learning from patterns in the data, allowing us to make accurate predictions based on these learned patterns.

### 2.6 Machine learning explainability with LIME

The local interpretable model-agnostic explanations (LIME) framework is a model-agnostic technique designed to investigate a ML model’s decision-making process on a per-instance basis (40, 64). LIME works by tweaking the input parameters of an already trained model while observing how these tweaks affect the model’s predictions. This process allows LIME to create a simplified, interpretable model that approximates the behaviour of the original complex model within a local region around a specific instance.

According to (65), LIME distinguishes itself from other interpretable models by centring its focus on providing explanations for individual predictions rather than attempting to elucidate the entirety of the model’s behaviour. In simpler terms, LIME adopts a localised approach instead of striving to explain the entire model. While numerous interpretable models aim to approximate the decision boundaries of a ML model globally, LIME recognises that understanding every facet of a complex model’s behaviour across all instances might be impractical. The mathematical formulation of LIME can be written as:

(1) $${\text{LIME}}_{\mathrm{explanation}}(x)=\arg\min_{g\in G}\left[L(f,g,\pi_{x})+\Omega (g)\right]$$

Where ${\text{LIME}}_{\mathrm{explanation}}(x)$ represents the explanation provided by LIME for the instance x. f is the original ML model whose predictions we want to explain. g is the local surrogate model, chosen from a family of interpretable models, G. $L(f,g,\pi_{x})$ is the loss function that quantifies the difference between the predictions of f and g, for instance, x, weighted by a proximity measure $\pi_{x}$. Ω(g) is a complexity penalty term that encourages simpler explanations provided by g. $\pi_{x}$ is a proximity measure that captures how similar an instance, z is to x, often using a kernel-based approach to define the neighbourhood around x. The minimisation process aims to find the best-fitting surrogate model, g, that balances prediction fidelity and interpretability.

### 2.7 Evaluation metrics

Evaluation metrics provide quantifiable measures to assess the performance of ML models in categorising data into different classes (66–68). These metrics offer insights into a model’s accuracy, precision, sensitivity, specificity, area under the curve (AUC), F1-score, and more.

(2) $$\text{Accuracy}=\frac{\text{TP}+\text{TN}}{\text{TP}+\text{TN}+\text{FP}+\text{FN}}$$

(3) $$\text{Precision}=\frac{\text{TP}}{\text{TP}+\text{FP}}$$

(4) $$\text{Sensitivity}\;(\text{recall})=\frac{\text{TP}}{\text{TP}+\text{FN}}$$

(5) $$\text{Specificity}=\frac{\text{TN}}{\text{TN}+\text{FP}}$$

(6) $$\text{F1 measure}=2\cdot \frac{\text{\textbackslash{},precision}\cdot \text{recall}}{\text{\textbackslash{},precision}+\text{recall}}$$

Understanding True Positive (TP), True Negative (TN), False Positive (FP), and False Negative (FN) is crucial for this study. TP denotes the model correctly identifying positive results, TN signifies the correct identification of negative results, FP represents incorrect positive labelling, and FN indicates a missed positive identification. For instance, a TP could involve accurately predicting graft failure, while a TN might involve identifying non-failure cases. Conversely, FP could misclassify graft failure, and FN might overlook actual failures.

The assessment of the model’s performance in the Kidney Graft Study encompasses several key metrics. Accuracy quantifies the overall correctness of predictions, reflecting both true positive and true negative rates. Precision gauges the ratio of true positive predictions to all positive predictions, emphasizing correctness in positive classifications. Sensitivity measures the model’s ability to correctly identify true positives, highlighting its effectiveness in capturing actual positive cases. Specificity evaluates the model’s aptitude in identifying true negatives, accentuating its proficiency in recognizing actual negative cases. The area under the curve (AUC) provides a comprehensive overview of the model’s discriminative power across varying thresholds. It elucidates the model’s ability to rank positive instances above negative ones. The F1-score harmonises precision and sensitivity, striking a balance between the two metrics. These metrics are pivotal for evaluating the model’s performance and drawing meaningful conclusions.

### 2.8 Permutation-based feature importance

We quantified the contribution of each feature in the predictive model using permutation-based feature importance (69–71) and graphically visualized the results using a combination of bar plot and box plot. This analysis employed Extra Tree models across different eras, assessing feature importance by permuting each feature and measuring the resulting change in model performance. Initially, model performance was measured using all features, referred to as the “full model performance.” Each feature’s values were then randomly permuted, and the model’s performance was reassessed. A feature was deemed “important” if permuting its values significantly increased the model’s prediction error, as indicated by an increase in 1−AUC, showing reliance on that feature. Conversely, a feature was considered “unimportant” if the permutation caused little change in 1−AUC, suggesting the model did not rely on that feature. Model performance was evaluated using 1−AUC as the loss function, with a larger increase indicating greater feature importance. To account for randomness in the permutation process, we computed the mean values of the loss function over 10 permutations. The bars’ lengths correspond to each feature’s average contribution or importance, while the boxplot represents the distribution and variability of each feature’s importance across different permutations. This approach quantified variability in feature importance and provided a robust ranking of feature contributions.

## 3 Results

This section presents the results of our experimental and model explanation studies. All experiments utilised the preprocessed data as outlined in Section 2. The experiment used the R (72) programming language, which is equipped with various statistical and graphical techniques for highly extensible machine-learning tasks. The experiments were performed on an AMD Threadripper 3990X 4.3 GHz GT 1,030 2 GB PRO high-performance workstation (288 MB Cache, 64x Cores, 128 Threads, 4.3 GHz Turbo), with an MSI TRX40 PRO 10G AMD Ryzen Threadripper motherboard, a GeForce RTX 2,070 8 GB GDDR6 graphics card a 3,200 MHz 64 GB gaming RAM, 1 TB M.2 SSD with up to 3.5 GB/s speed, and a 4TB HDD. This device provided the computational power necessary to handle the various stages of data preprocessing, model building, and evaluation involved in the analysis.

### 3.1 Performance evaluation of the classifiers

**Figure 3:**
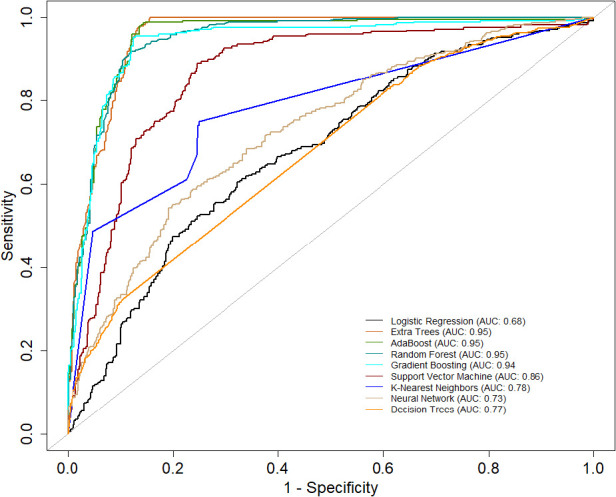
AUC of the classifiers for the *pre-CYA* era.

Table 2 presents a comprehensive overview of model performance during the *pre-CYA era*, complemented by the ROC curve depicted in Figure 3. These results offer valuable insights into the effectiveness of the models. The ensemble classifiers exhibited superior performance, boasting an AUC of 94% and above and an Accuracy of 86% and above. Notably, the AdaBoost model demonstrated particularly high performance across several evaluation metrics. Conversely, the Logistics regression model showed relatively lower performance in the *pre-CYA* era. The enhanced performance of ensemble classifiers can be attributed to their adeptness in mitigating overfitting and effectively handling noisy data. This resilience positions them as a robust choice for this particular era.

**Table 2:** Performance evaluation of the models on the *pre-CYA*.

Model	Accuracy	Precision	AUC	F1	Sensitivity	Specificity
Logistic regression	0.65	0.61	0.68	0.49	0.40	0.82
Extra Trees	0.86	0.87	0.95	0.82	0.78	0.92
AdaBoost	0.87	0.88	0.95	0.83	0.78	0.93
Random Forest	0.86	0.89	0.95	0.81	0.75	0.93
Gradient Boosting	0.86	0.89	0.94	0.81	0.75	0.94
Support Vector Machine	0.79	0.75	0.86	0.75	0.75	0.83
K-Nearest Neighbors	0.71	0.66	0.78	0.63	0.61	0.78
Neural network	0.69	0.65	0.73	0.60	0.56	0.78
Decision Trees	0.73	0.69	0.77	0.66	0.63	0.80

**Figure 4:**
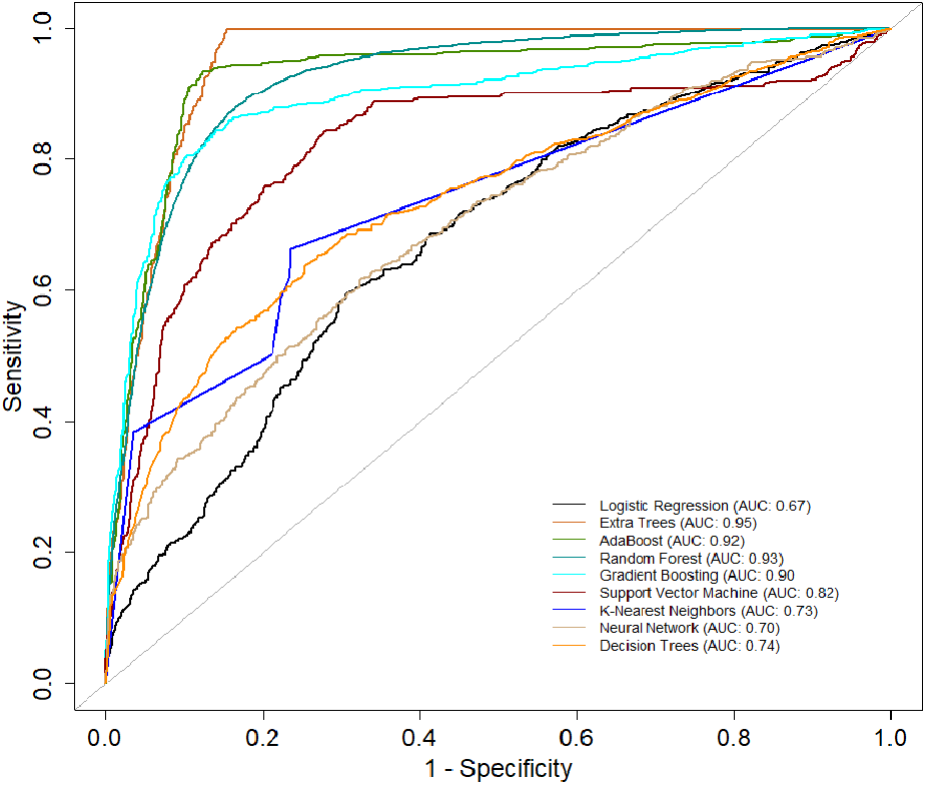
AUC of the classifiers for the CYA era.

Table 3 presents another model performance during the *CYA* era. The ensemble classifiers also performed the best overall, with Extra Trees showing the highest scores in AUC (0.95), accuracy (0.84), and sensitivity (0.68). At the same time, Logistic regression had the lowest scores across all metrics except for specificity, where it scored 0.87, which was higher than K-Nearest Neighbors and Decision Trees. Figure 4 presents the ROC curve of the classifiers in the *CYA* era.

**Table 3:** Performance evaluation of the models on the *CYA* era.

Model	Accuracy	Precision	AUC	F1	Sensitivity	Specificity
Logistic regression	0.66	0.55	0.67	0.38	0.29	0.87
Extra Trees	0.84	0.85	0.95	0.75	0.68	0.93
AdaBoost	0.83	0.86	0.92	0.74	0.65	0.94
Random Forest	0.82	0.86	0.93	0.70	0.58	0.95
Gradient Boosting	0.84	0.87	0.90	0.75	0.65	0.95
Support Vector Machine	0.79	0.77	0.82	0.68	0.60	0.90
K-Nearest Neighbors	0.69	0.57	0.73	0.54	0.50	0.79
Neural network	0.69	0.61	0.70	0.47	0.38	0.87
Decision Trees	0.73	0.65	0.74	0.59	0.54	0.84

**Figure 5:**
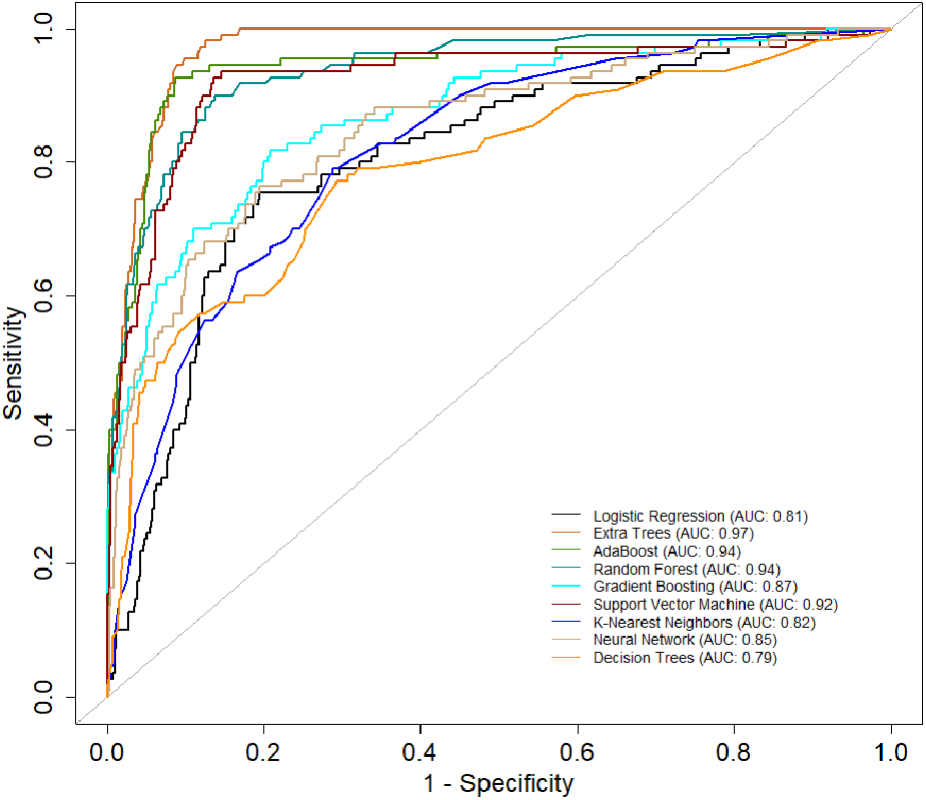
AUC-ROC of the classifiers for the New-Gen.

Table 4 provides an evaluation of model performance in the *New-Gen* era. Based on the AUC score and other metrics listed in the table, Extra Trees outperforms other models, followed by Random Forest, AdaBoost, and SVM. Logistic Regression and Decision Trees performed the worst according to the AUC score. The high specificity values across all models indicate their effectiveness in identifying true negatives. Figure 5 presents the ROC curves and AUC scores of the different classifiers in the *New-Gen* era.

**Table 4:** Performance evaluation of the models on the New-Gen.

Model	Accuracy	Precision	AUC	F1	Sensitivity	Specificity
Logistic regression	0.84	0.42	0.81	0.42	0.15	0.96
Extra Trees	0.92	0.82	0.97	0.82	0.59	0.98
AdaBoost	0.90	0.80	0.94	0.80	0.50	0.98
Random Forest	0.91	0.84	0.94	0.84	0.49	0.98
Gradient Boosting	0.89	0.64	0.87	0.74	0.45	0.97
Support Vector Machine	0.91	0.74	0.92	0.84	0.48	0.98
K-Nearest Neighbors	0.85	0.84	0.82	0.62	0.15	0.98
Neural network	0.89	0.62	0.85	0.71	0.45	0.97
Decision Trees	0.88	0.71	0.79	0.64	0.46	0.95

### 3.2 Model comparisons and historical transportability assessment

**Figure 6:**
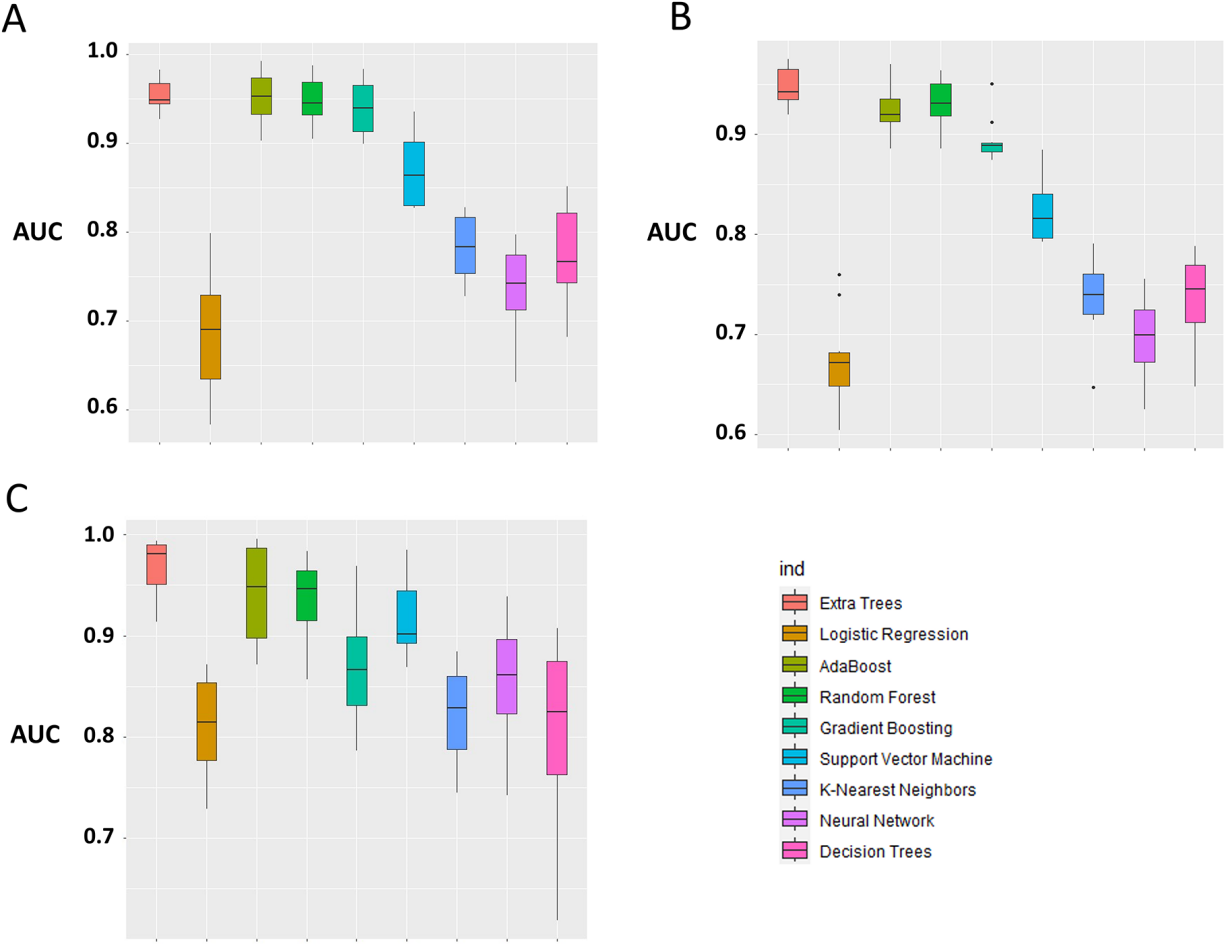
Box plot of model performance evaluation on the test-folds, based on the distribution of the AUC for each model. The Extra Trees was used as the benchmark for comparison with other models. (**A**) *pre-CYA*, (**B**) *CYA* and (**C**) *New-Gen*. The significant difference was based on the Wilcoxon Signed-Rank Test at a 5% level of significance.

**Figure 7:**
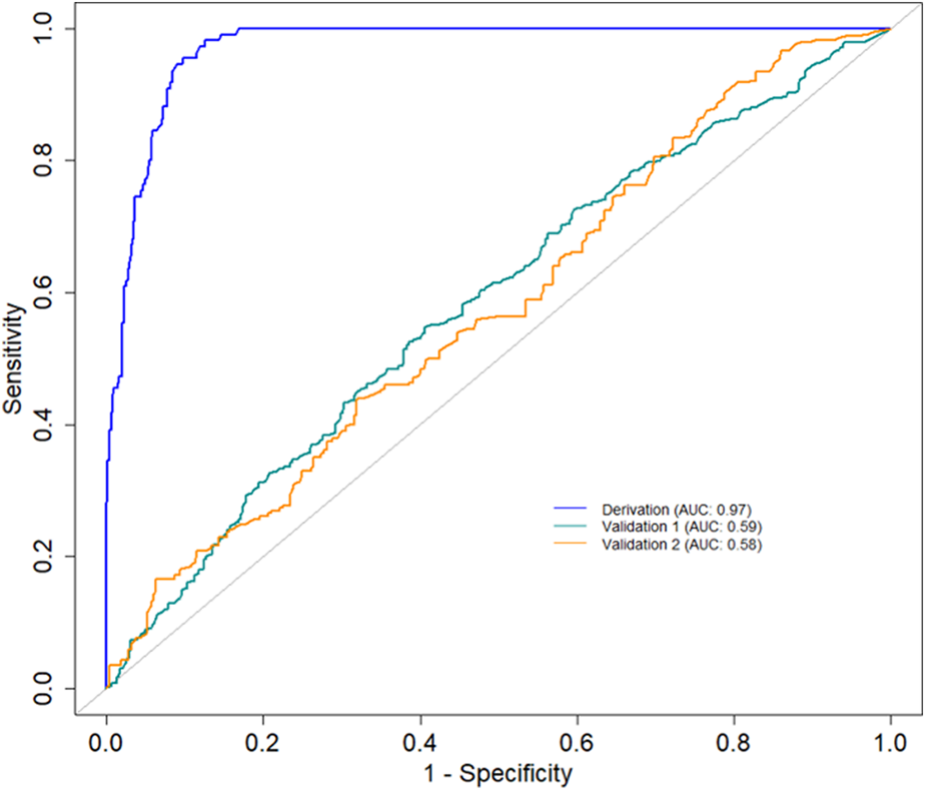
AUC curves demonstrating the historical transportability of the Extra Tree model in the *New-Gen* (Derivation) to the *CYA* (Validation 1) and *pre-CYA* (Validation 2).

The primary evaluation metric shows that the Ensemble classifiers, especially the Extra trees algorithm, are the best-performing models across the three eras. We further evaluated the validity of this claim by assessing whether the observed differences in the model performances are statistically significant. Statistical significance was ascertained using the Wilcoxon signed-rank test at a 5% significance level. Figure 6A shows that the Extra Trees model had a narrower range in the AUC scores for the 10-fold CV compared to other models. It is shown that the distribution of the Extra Trees model significantly differs from the non-ensemble classifiers. Comparing the model performance distribution in *CYA* and *New-Gen* (Figures 6B,C), there are statistical differences between the Extra Trees and other models, except for Random Forest in the *CYA* era. As the experimental results confirmed the reproducibility of the selected models using the re-sampling technique, we further assessed the transportability of the *New-Gen* model to other eras (Figure 7). The external validation of the Extra Trees model in the *New-Gen* era shows a lower discriminative power in the *CYA* (AUC: 0.59) and *pre-CYA* (AUC: 0.58) eras.

### 3.3 Explanation of the outcomes

**Figure 8:**
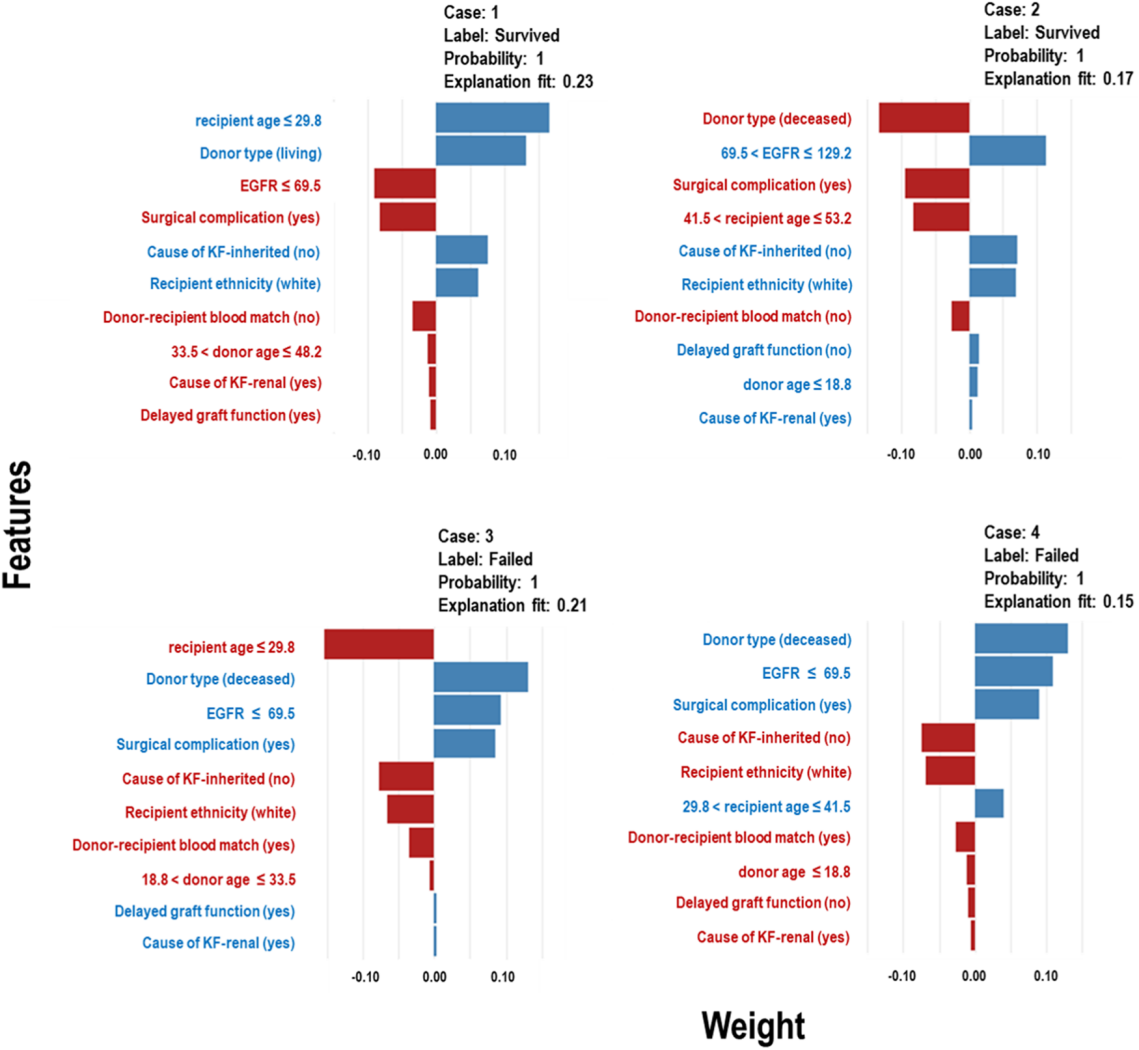
LIME model plots explaining individual predictions for four randomly selected patients who underwent transplants in the *pre-CYA* era. The plots are based on the Extra Tree model and show the features that support (blue bars) or contradict (red bars) the predicted probability.

**Figure 9:**
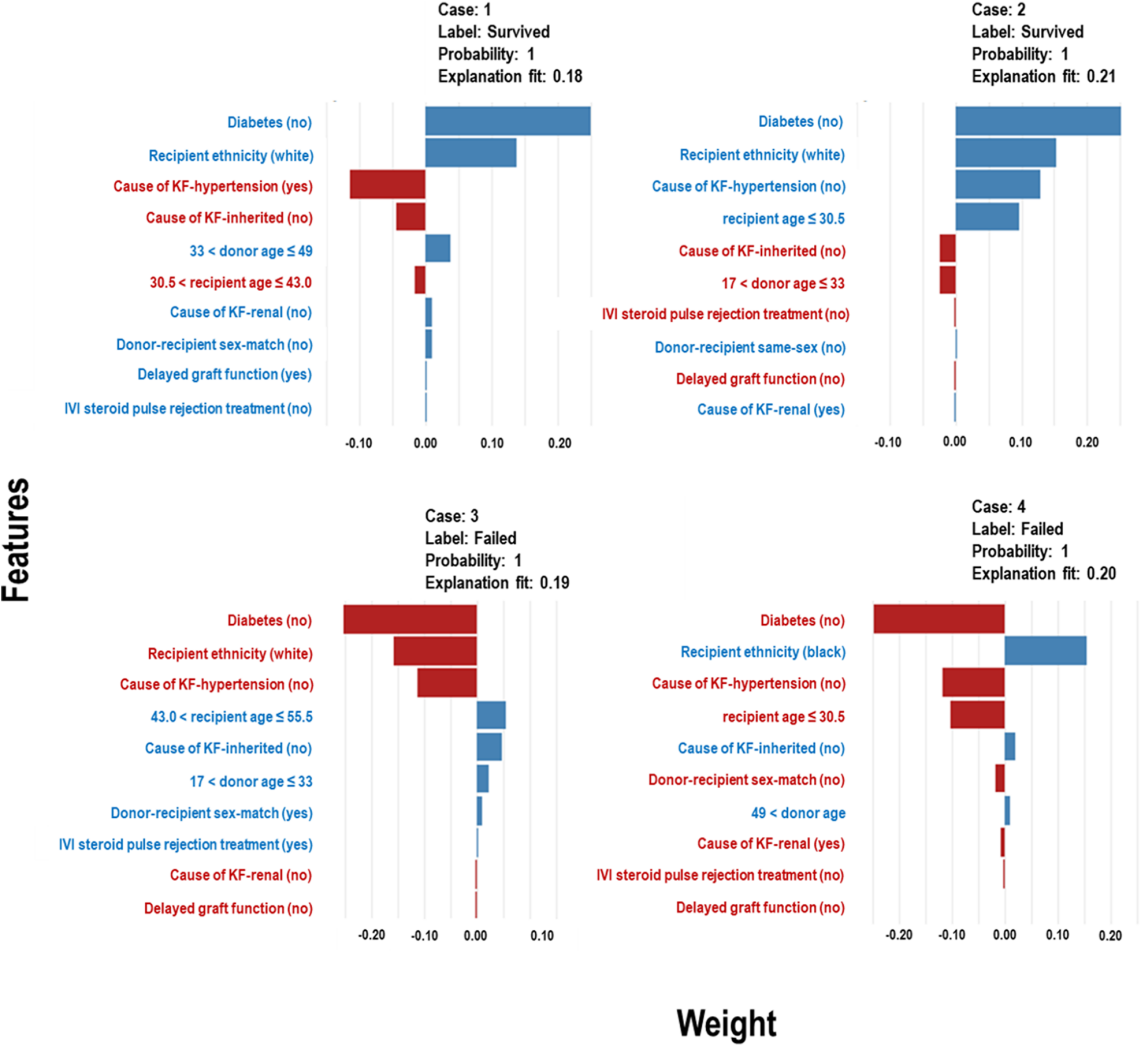
LIME model plots explaining individual predictions for four randomly selected patients who underwent transplants in the *CYA* era. The plots are based on the Extra Tree model and show the features that support (blue bars) or contradict (red bars) the predicted probability.

**Figure 10:**
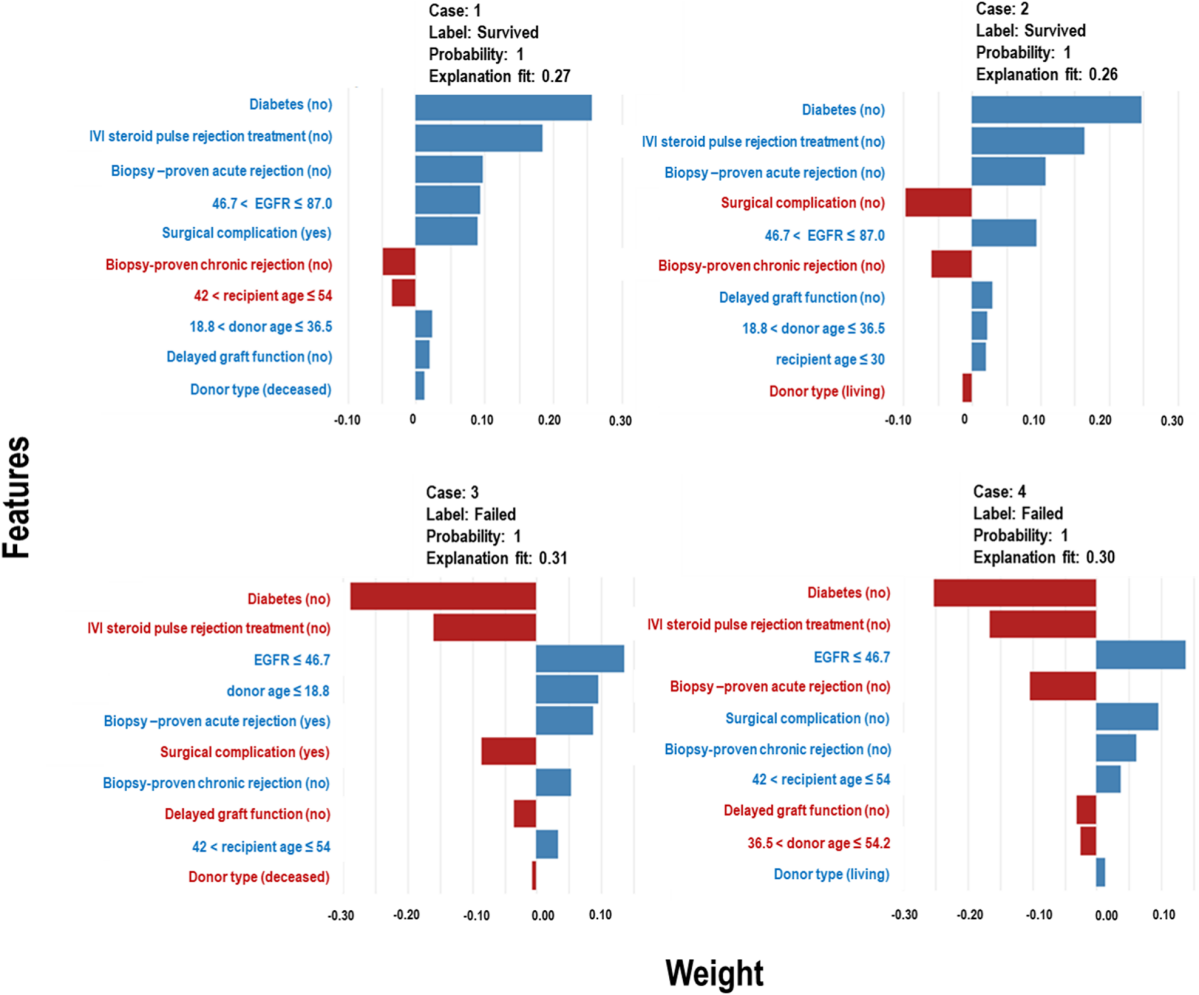
LIME model plots explaining individual predictions for four randomly selected patients who underwent transplants in the *New-Gen* era. The plots are based on the Extra Tree model and show the features that support (blue bars) or contradict (red bars) the predicted probability.

The Extra Trees classifier was chosen to develop the LIME model across all three eras to ensure interpretability and transparency. The LIME model is a valuable tool for interpreting predictions from any classifier in an understandable and interpretable manner. A feature importance bar plot was employed to elucidate predictions in the local region. Visual representations of the LIME results are presented in Figures 8, 9, and 10, where blue and red colours signify contributing factors. Specifically, blue denotes features that increase the likelihood of graft survival or failure. At the same time, red indicates features that negatively influence the likelihood of graft survival or failure. The length of the bars in the LIME summary plot indicates the magnitude of a feature’s influence on the model’s prediction for that particular case, with longer bars representing a more significant impact and shorter bars representing minimal influence. Understanding these contributing factors is paramount for identifying potential risks and making informed patient care and treatment planning decisions. The figures display predicted probabilities assigned by the Extra Tree model for each sample case categorised as “Survived” or “Failed”. Additionally, the explanation fit indicates how well the interpretable model approximates the behaviour of the underlying Extra Tree model for both the “Survived” and “Failed” classes. These values are explicitly presented in each figure.

**Figure 11:**
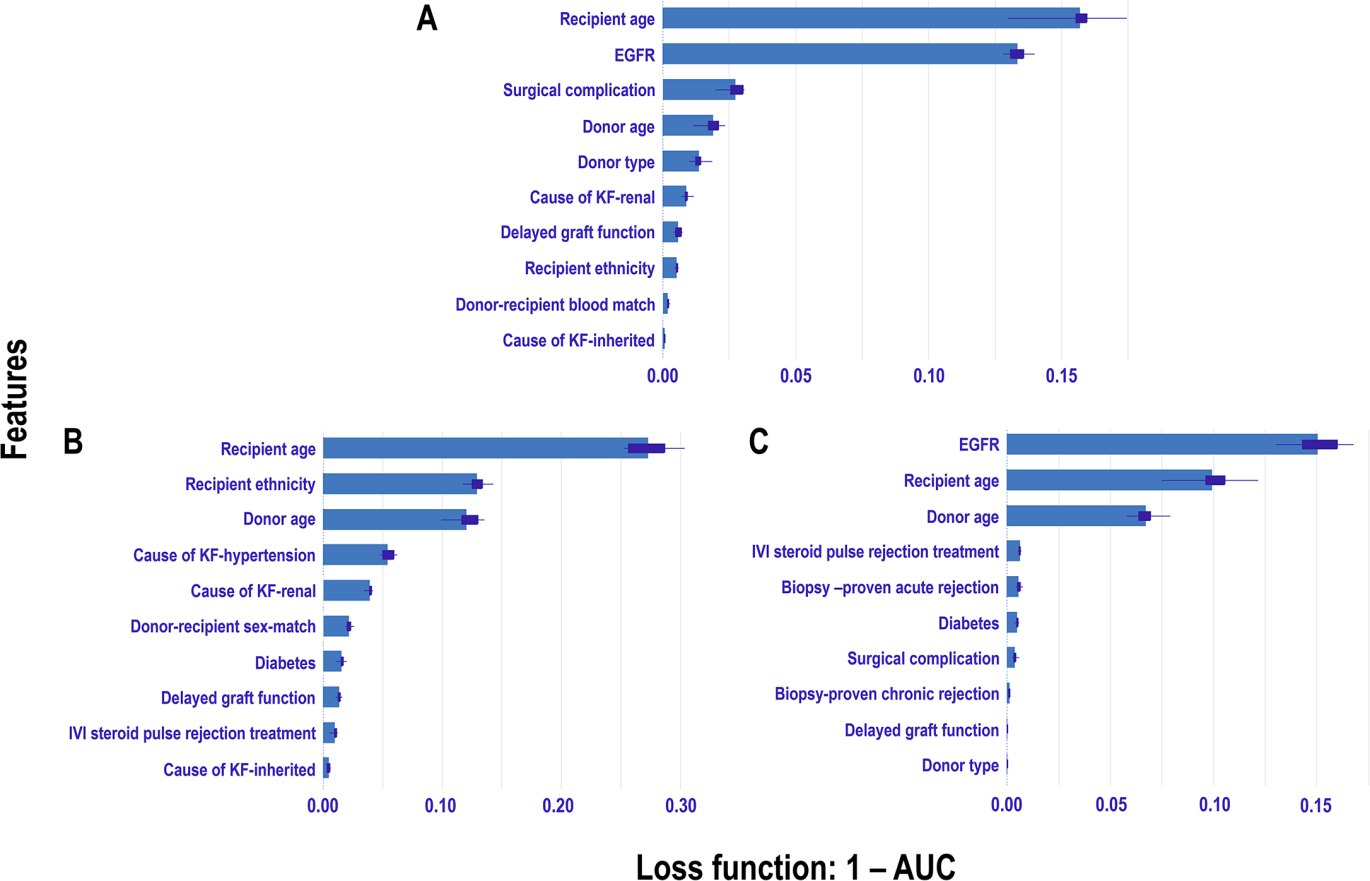
Permutation-based feature importance measures across eras: The plot displays the permutation-based feature importance measures for study features included in the Extra Trees models for each era: **A** = *Pre-CYA*, **B** = *CYA*, and **C** = *New-Gen*. Feature importance is measured using *1-AUC* as the loss function, where higher values indicate greater impact on model performance when the feature is permuted.

Figure 8 presents the LIME results for the *Pre-CYA* era, showcasing the influence of different factors on graft survival or failure in four randomly selected patients. In Figure 8 (Case 1), factors that negatively influenced graft survival, include low estimated glomerular filtration rate (EGFR), presence of surgical complication, unmatched donor-recipient blood group type and donors aged approximately 34 to 48 years or deceased donors. Conversely, being a younger recipients or white recipient and having no inherited cause of KF contributed positively to graft survival. The influence of these features on graft survival follows a similar pattern in Case 2, even when considering different categories of the features. In Figure 8 (Cases 3), contributing factors to graft failure include non-living donors, low EGFR, surgical complication, DGF and presence of primary renal cause of KF. Similar trend and patterns are observed in Figure 8 Case 4.

In Figure 9 (Cases 1 and 2), the model identifies several factors that increase the likelihood of graft survival. These include being a non-diabetic or white recipient, being a younger recipient, receiving a kidney from donors between the ages of 33 and 49, and not having hypertension as a cause of KF. Other factors, such as delayed graft function (DGF), that positively influence survival may not be significant, as shown in the plot. Conversely, in cases of graft failure (Figure 9 - Cases 3 and 4), factors contributing to graft failure include being a black recipient, being a recipient between 43 and 56 years, not having an inherited cause of KF, and receiving kidneys from donors aged approximately 17 to 33 years.

In Figure 10, Cases 1 and 2, classified as “Survived,” and Cases 3 and 4, classified as “Failed” by the Extra Tree model in the *New-Gen* era, are depicted. The figure illustrates that specific factors, such as being a non-diabetic recipient, not receiving IVI steroids as rejection treatment, the absence of acute rejection, having a high EGFR, or experiencing surgical complications, as well as other feature categories shown in the blue bars, positively influence graft survival in Cases 1 and 2. Conversely, in Cases 3 and 4, we observed the reverse effects of these features on graft failure.

### 3.4 Feature importance

For the *pre-CYA* era (Figure 11A), the feature importance plot shows recipient age and EGFR are the most significant features, with recipient age demonstrating the highest importance for predicting graft failure in this era. Notably, EGFR shows a narrower boxplot than recipient age, suggesting it has less variability and a more stable contribution to the model’s predictions. Meanwhile, features such as surgical complication, donor age, and donor type contribute moderately to the model prediction, whereas other features including primary renal causes of KF, DGF and recipient ethnicity have minimal influence on predictions. The recipient age feature is the most influential in the *CYA* era (Figure 11B), exhibiting the highest mean importance and moderate variability. Recipient ethnicity and donor age are also significant contributors. The other features, such as hypertension or renal as a cause of KF, have moderate importance with relatively low variability. In contrast, DGF , IVI steroids as rejection treatment and inherited cause of KF have minimal impact on model performance. For the *New-Gen* era (Figure 11C), the feature importance plot shows that EGFR, recipient age, and donor age are the most influential features, with EGFR showing the highest mean importance. Features such as IVI steroids as rejection treatment and biopsy-proven acute rejection and diabetes also contribute to the model, though with less significance. The remaining features, including biopsy-proven chronic rejection, DGF, and donor type, have minimal influence on the model’s predictive capabilities.

## 4 Discussion

This study evaluates nine machine learning (ML) models to predict the 10-year risk of graft failure after kidney transplantation. Advances in medical practices, including immunosuppressive drugs and supportive therapies, have positively impacted graft survival post-transplant. Exploratory data analysis revealed substantial improvement in graft survival rates from the *pre-CYA* to the *New-Gen* transplant eras, indicating reduced risk of graft failure over time. Specific patient or donor characteristics influencing graft survival have also improved, leading us to hypothesise varying prognostic factors across the three transplantation eras and prompting the modelling of graft failure during each era. We aimed to develop an optimisable platform for predicting graft failure post-transplant, with adaptable methodological strategies for future studies using more recent data to identify risk factors and support clinical decision-making (34).

We internally validated nine models for each transplant era, both with and without data augmentation. Given the study’s relatively small sample size, we addressed potential issues related to model reproducibility and overfitting. Results indicate that all nine selected algorithms demonstrated good discrimination ability measured by the AUC metric. Ensemble algorithms consistently outperformed others in predicting graft failure, benefitting from additional samples and diversity introduced by augmented data (Table A1), aligning with studies emphasising the importance of large datasets for accurate ML (73). While direct comparison with prior studies modelling era-specific graft survival in kidney transplants was not possible, our best-performing models achieved an AUC score of 97%, which is comparable to or higher than AUC scores reported in studies modelling long-term graft survival, which ranged from 64.4% to 89.7% (30, 32, 74–77). As depicted in Table 5, the variation in these scores among studies can be attributed to several factors, including differences in data size, study periods, risk associations, and modeling strategies.

**Table 5:** Comparison with other existing studies.

Reference	Method	Accuracy	AUC	Sensitivity	Specificity
Yoo et al. (77)	Decision Tree Ensemble	80.0	–	–	–
Pahl et al. (74)	Random Forest	–	64.4	–	–
Badrouchi et al. (30)	Multiple ML models	91.5	89.7	91.9	87.5
Naqvi et al. (32)	Multiple ML models	–	82.0	–	–
Salaun et al. (76)	RF and other models	–	84.4	–	–
Paquette et al. (75)	Artificial Neural Networks	66.1	–	–	–
**Our Study**	**Extra Trees, together with other models**	**92.0**	**97.0**	**78.0**	**98.0**

Top features influencing graft survival showed inconsistency across the three eras, except for recipient and donor age, which consistently demonstrated global importance. This highlights the variation in graft survival factors across eras. LIME models provided interpretable results for features influencing graft survival within each era, emphasizing the necessity for continuous adaptation and validation of predictive models in different contexts. Incorporating interpretable ML models like LIME into clinical decision-making can lead to more informed and individualized treatment plans, improving patient outcomes and graft survival rates (40, 64).

Our study also evaluated the transportability of the *New-Gen* model to other eras, revealing challenges due to changing disease severity over time (28). Differences in survival rates and risk factors across the three eras indicate that historical transportability may only be achieved if the same features consistently impact graft survival. Despite these challenges, our models exhibited reproducibility and consistency in predicting outcomes within each era, underscoring the potential of ML approaches to enhance understanding and prediction of graft survival across diverse settings.

In conclusion, this study concurrently explores graft survival across three transplant eras, providing valuable insights into post-kidney transplant outcomes. Acknowledging limitations, including reliance on data from a single centre with a relatively small patient cohort, is crucial. While findings may not fully capture the entire landscape or current state of kidney transplants in South Africa, they provide a foundation for future studies.

Further ML-based investigations into graft survival, utilising current data from diverse regions, are essential to deepen our understanding. The study’s comprehensiveness could have been enhanced by incorporating pivotal variables, which, unfortunately, were excluded due to missing data or inconsistencies in the data collection process.

Looking forward, our objective is to refine and assess the geographical applicability of the models developed within a different transplant unit in South Africa, with a primary focus on improving transportability within the same transplant era.

## Data Availability

The data analyzed in this study is subject to the following licenses/restrictions: Data can be shared on request. Requests to access these datasets should be directed to okechinyere.achilonu@wits.ac.za.

## Appendix A

Additional results Appendix A.

**Table A1:** Results from the analysis taken without SMOTE.

Era	Model	Accuracy	Precision	AUC	F1	Sensitivity	Specificity
New-Gen	Logistic regression	0.84	0.31	0.73	0.12	0.07	0.97
	Extra Trees	0.84	0.45	0.75	0.37	0.31	0.93
	AdaBoost	0.83	0.40	0.75	0.32	0.27	0.93
	Random Forest	0.85	0.48	0.73	0.32	0.24	0.96
	Gradient Boosting	0.85	0.52	0.73	0.34	0.26	0.96
	Support Vector Machine	0.85	0.54	0.70	0.21	0.13	0.98
	K-Nearest Neighbors	0.84	0.25	0.75	0.03	0.02	0.99
	Neural network	0.84	0.45	0.70	0.31	0.24	0.95
	Decision Trees	0.83	0.41	0.64	0.33	0.27	0.93
CYA	Logistic regression	0.66	0.56	0.66	0.38	0.29	0.87
	Extra Trees	0.61	0.45	0.58	0.42	0.40	0.73
	AdaBoost	0.59	0.42	0.57	0.40	0.38	0.70
	Random Forest	0.64	0.47	0.63	0.20	0.13	0.92
	Gradient Boosting	0.66	0.55	0.66	0.40	0.32	0.85
	Support Vector Machine	0.65	0.55	0.64	0.22	0.14	0.94
	K-Nearest Neighbors	0.63	0.46	0.60	0.26	0.18	0.88
	Neural network	0.67	0.59	0.66	0.33	0.23	0.91
	Decision Trees	0.58	0.39	0.56	0.34	0.31	0.73
pre-CYA	Logistic regression	0.60	0.52	0.65	0.45	0.40	0.74
	Extra Trees	0.64	0.57	0.66	0.56	0.56	0.70
	AdaBoost	0.61	0.53	0.67	0.51	0.49	0.70
	Random Forest	0.62	0.54	0.66	0.52	0.50	0.70
	Gradient Boosting	0.62	0.54	0.66	0.53	0.52	0.69
	Support Vector Machine	0.60	0.53	0.65	0.44	0.37	0.77
	K-Nearest Neighbors	0.61	0.53	0.65	0.57	0.61	0.62
	Neural network	0.62	0.56	0.67	0.51	0.47	0.73
	Decision Trees	0.60	0.52	0.62	0.50	0.47	0.69
